# Spatiotemporal Dynamics of Surface Ozone and Its Relationship with Meteorological Factors over the Beijing–Tianjin–Tangshan Region, China, from 2016 to 2019

**DOI:** 10.3390/s22134854

**Published:** 2022-06-27

**Authors:** Linyan Bai, Jianzhong Feng, Ziwei Li, Chunming Han, Fuli Yan, Yixing Ding

**Affiliations:** 1 International Research Center of Big Data for Sustainable Development Goals, Beijing 100094, China; baily@radi.ac.cn (L.B.); liiiziwei@163.com (Z.L.); hancm@radi.ac.cn (C.H.); yanfl@radi.ac.cn (F.Y.); dingyx@radi.ac.cn (Y.D.); 2 Key Laboratory of Digital Earth Science, Aerospace Information Research Institute, Chinese Academy of Sciences, Beijing 100094, China; 3 Agricultural Information Institute, Chinese Academy of Agricultural Sciences, Beijing 100081, China; 4 College of Geometics, Xi’an University of Science and Technology, Xi’an 710054, China

**Keywords:** surface ozone, OMI, spatiotemporal dynamics, meteorological factors

## Abstract

In recent years, ozone pollution has been increasing in some parts of the world. In this study, we used the Beijing–Tianjin–Tangshan (BJ-TJ-TS) urban agglomeration region as a case study and used satellite remotely sensed inversion data and hourly ground monitoring observations of surface ozone concentrations, meteorological data, and other factors from 2016 to 2019 to explore the spatiotemporal dynamic characteristics of surface ozone concentration and its pollution levels. We also investigated their coupling relationships with meteorological factors, including temperature, pressure, relative humidity, wind velocity, and sunshine duration, in order to support the development of effective control measures for regional ozone pollution. The results revealed that the surface ozone concentration throughout the BJ-TJ-TS region from 2016 to 2019 exhibited an overall pattern of high values in the northwest and low values in the southeast, as well as an obvious difference between built-up and non-built-up areas (especially in Beijing). Meanwhile, a notable increasing trend of ozone levels was discovered in the BJ and TJ areas from 2016 to 2019, whereas this upward trend was not evident in the TS area. In all three areas, the highest monthly average ozone values occurred in the summer month of June, while the lowest monthly average levels occurred in the winter month of December. Their diurnal variation values reached a maximum value at approximately 3:00–4:00 p.m. and a minimum value at approximately 7:00 a.m. It is clear that high temperature, long sunshine duration, low atmospheric pressure, and weak wind velocity conditions, as well as certain relative humidity levels, readily led to high-concentration ozone pollution. Meanwhile, the daily average values of the five meteorological factors on days with Grade I and Grade II ozone pollution displayed different characteristics.

## 1. Introduction

Ozone, which mainly exists in the lower level of the stratosphere (i.e., approximately 90% from 20 to 25 km above sea level, with the remaining 10% in the troposphere), protects life on Earth from harmful ultraviolet (UV) radiation from the Sun reaching the surface [1]. Unlike natural stratospheric ozone, surface ozone is considered to be an important gaseous pollutant. High concentrations of ozone near the surface can be harmful to humans, animals, crops, and other organisms and materials [2,3,4]. Surface ozone is created through the photochemical interactions of man-made (and natural) emissions of volatile organic compounds (VOCs), nitrogen oxides (NOx), methane (CH4), and free radicals (HOx = OH + HO2 + RO2) in the presence of heat and sunlight [5]. In recent years, ozone pollution, which has attracted extensive attention, has become an important subject and challenge in the prevention and control of atmospheric environmental pollution. Correspondingly, the spatial and temporal distribution characteristics of ozone, as well as the correlation analysis with influencing factors, have become hot topics in air pollution research.

In many parts of the world, as a result of rapid economic development and urbanization, excessive emissions of atmospheric (air) pollutants, derived from accelerated industrial development and huge increases in the number of vehicles, have occurred. These emissions, along with the effects of global warming and other natural and artificial factors, have resulted in the continuous increase in the world’s local surface ozone concentration [6,7,8]. For example, some urban metropolitan areas, agglomeration regions, and huge industrial zones with high population densities and intense human activities have become emission-source areas of high-concentration ozone as well as influential areas of ozone pollution, such as the urban agglomerations in the Yangtze River Delta and Pearl River Delta in China [9], the urban belts along the east and west coasts of the United States [10,11], and some of the urban agglomerations in Western Europe [12]. Meanwhile, the distribution of and variation in surface ozone concentration in those areas vary greatly in geographical time and space. The driving factors, including various precursor emissions, changes in meteorological variables, differences in background conditions, patterns of ozone and boundary layer structures, regional forest coverage rates, and even urban heat island effects, display specific characteristics in each area [13,14,15,16,17,18]. These factors form their own complex spatiotemporal internal interaction mechanisms and, simultaneously, exert considerable comprehensive influences on ozone formation and the occurrence of ozone pollution in local and adjacent areas, as well as larger regions.

In recent years, China has adopted a series of policies and measures to improve air quality [19]. With the decrease in PM_2.5_, surface ozone has now become a serious environmental pollutant in many cities in China [6]. The Beijing–Tianjin–Tangshan (BJ-TJ-TS) urban agglomeration region, as one of the four industrial bases in China and one of the country’s largest, most densely populated, and most economically developed urban agglomerations, is experiencing increasingly serious ozone pollution. Limited by spatial distribution, some site-based studies have not been able to fully analyze the spatial distribution of ozone pollution, especially in the urban agglomeration.

**Figure 1:**
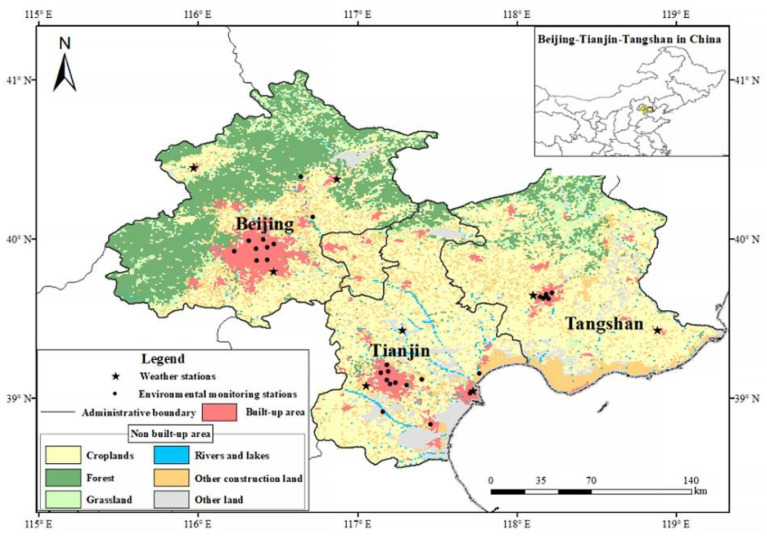
Study region and ground-based observation stations.

Given this limitation, in this study, the temporal and spatial distribution characteristics of surface ozone in the BJ-TJ-TS region of China (see Figure 1) from 2016 to 2019 were analyzed using the surface ozone concentration data retrieved from Ozone Monitoring Instrument (OMI) remote sensing data, as well as surface air quality and meteorological station data. By analyzing the correlation between meteorological conditions and ozone concentration, the differences between the meteorological conditions affecting ozone concentration in built-up and non-built areas were explored.

## 2. Data and Methods

### 2.1. Data

The ozone concentration data were obtained from observed urban air quality data released in real time by the China National Environmental Monitoring Center (http://www.cnemc.cn/ accessed on 1 March 2020). Hourly ozone concentration data from 2016 to 2019 were collected from 28 stations (represented by the black dots in Figure 1) to determine the monthly, seasonal, and annual averages. To ensure the accuracy of the research results, data quality control was carried out in accordance with the data validity requirements in the Environmental Air Quality Standard (GB3095-2012). Surface ozone concentration datasets (SOCDs) derived from the teamwork of the Aerospace Information Research Institute (AIRI) of the Chinese Academy of Sciences and its partners, which were published in 2021 [20], over the BJ-TJ-TS area were employed. Meanwhile, five meteorological parameters, namely, pressure (hPa), temperature (°C), relative humidity (%), wind speed (ms^−1^), and sunshine duration (h), were collected from the three weather stations located in the study area (represented by the black stars in Figure 1), distributed by the National Meteorological Data Center of China (http://data.cma.cn/ accessed on 10 March 2020).

### 2.2. Analyses

Owing to surface ozone (concentration) as a geo-element field, the spatiotemporal pattern becomes one important component of its geospatial characteristics, which is the basis of analyzing and presenting the geospatial differentiation and dynamic evolution of surface ozone in geographic space. The remote sensing survey is a burgeoning approach being more and more widely used in various geographical aspects, whose quantitative remote sensing inversion is very promising for expanding and deepening its related application domains [21]. In this study, we used the SOCD data from 2016 to 2019, which were reversed from satellite remotely sensed tropospheric total ozone column data based on an optimal back-propagation (BP) neural network through multi-model optimization selection and has thus gone through strict data quality control procedures by using systematic validation and error precision analyses [20], to explore the study area’s spatial distribution of and temporal dynamic changes in surface ozone concentration [22].

Henceforth, the interannual variations in surface ozone concentration from 2016 to 2019 were analyzed using the Mann–Kendall (MK) test [23,24]. The nonparametric MK trend test is widely used to assess the significance of monotonic trends in time series, such as rainfall and evaporation. To convey increasing or decreasing expressions, the Mann–Kendall *Z_MK_* value is taken into consideration. The decreasing (increasing) expression is used for the time series with negative (positive) *Z_MK_* values. The standard *Z_MK_* value for the MK test is expressed as

(1) $$Z_{MK}=\{\begin{array}{c} \frac{S-1}{V\left(S\right)}\;for\;S>0\\ 0\;\;\;for\;S=0\\ \frac{S+1}{V\left(S\right)}\;for\;S<0 \end{array}$$

(2) $$V\left(S\right)=\frac{n\left(n-1\right)\left(2n+5\right)}{18}$$

(3) $$S=\sum_{k=1}^{n-1}\sum_{j=k+1}^{n}sgn\left(x_{j}-x_{k}\right)$$

(4) $$sgn\left(x_{j}-x_{k}\right)=\{\begin{array}{c} +1\;if\;\left(x_{j}-x_{k}\right)>0\\ 0\;\;if\;\left(x_{j}-x_{k}\right)=0\\ -1\;if\;\left(x_{j}-x_{k}\right)<0\; \end{array}$$

where *V*(*S*) is the variance, and *S* is the Kendall sum statistic. The differences between consecutive values are calculated so as to depict positive (+1), negative (−1), and neutral (0) signs, *sgn*(·) in Equation (4), where *x_j_* and *x_k_* are time series values at time instances *j* and *k*, respectively, from a given time series [1, *x_k_*, …, *x_j_*, …, *x_n_*] with *n* observations.

To quantitatively analyze the correlation between surface ozone concentration and meteorological elements (temperature, pressure, wind speed, sunshine duration, and relative humidity), the Pearson correlation coefficient (Spearman correlation coefficient) was utilized to measure the association between surface ozosne concentration and meteorological elements, which fit the normal distribution (abnormal distribution).

## 3. Results and Discussion

### 3.1. Spatiotemporal Dynamic Features of Surface Ozone Concentrations

**Figure 2:**
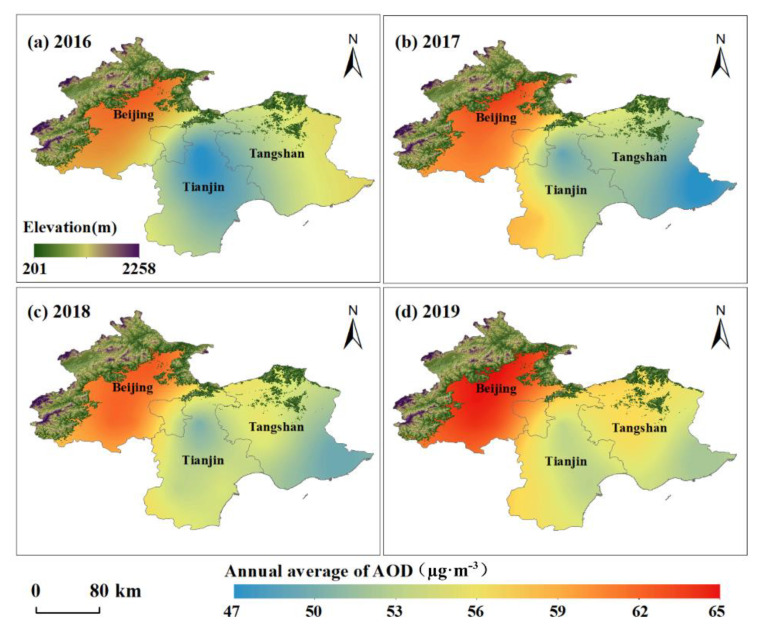
Spatial distributions of surface ozone average concentration in the BJ-TJ-TS region from 2016 to 2019.

As is known to all, spatial and temporal (spatiotemporal) dynamics is the most basic way to describe a field. Thus, for surface ozone (concentration), a geo-element field (also called a surface state field) exploring its spatiotemporal distribution and dynamic change characteristics and/or laws in geographical space is one of the main objectives and tasks of discovering its attributes, which is also an apparent approach for probing into other relevant questions (such as attribution) [25]. As shown in Figure 2, the surface ozone concentration in the BJ-TJ-TS region exhibited an overall pattern of high values in the northwest and low values in the southeast from 2016 to 2019, and there was an especially high-value part over Beijing’s central and north-central areas, which is consistent with the results of in situ (field) surveys (that were implemented from the northwest suburb to downtown areas of Beijing by Zhang et al. [26]). During this period, it is obvious that there was a large difference in the surface ozone concentration levels, as well as their change rates, between the built-up and non-built-up areas of the whole study region (BJ-TJ-TS), the former of which had a slow upward trend (with a value of *Slope* = 1.599), and in contrast, the latter had a fast upward trend (with a value of *Slope* = 3.712). In particular, the ozone concentration level (with an interannual average value of 57.18 µg·m^−3^) over the built-up area of BJ differed more notably from that of 61.59 µg·m^−3^ over the non-built-up area of BJ, while its increasing trend (with rate values of *Slope* = 0.775 and *β_slope_* = 0.026) in the former was more significantly slower than that of the latter (with values of *Slope* = 2.45 and *β_slope_* = 0.082) (though it did not pass the significance test) (Table 1 and Table 2). Additionally, there was a dynamic change in surface ozone concentration between Beijing’s built-up and non-built-up areas, which was similar to the whole BJ-TJ-TS area. In 2016, the surface ozone concentration over the built-up area of BJ was higher than that of the non-built-up area of BJ, whereas from 2017 to 2019, the ozone concentration over the non-built-up area exceeded that of the built-up areas.

As for Tianjin (TJ), from 2016 to 2019, there were generally minor differences between the surface ozone concentration distributions over the built-up and non-built-up areas, especially in the central and northern areas, as well as the Binhai New Area, in which they were slightly lower (see Figure 2), which may be related to the relatively high content of other atmospheric pollutants (e.g., CO, SO_2_, and PM_2.5_ fine particulate pollutants) [27,28]. Additionally, over this four-year study period, the surface ozone concentration level (with an interannual average value of 60.89 µg·m^−3^) over the built-up area of TJ was higher than that of the non-built-up area of TJ (with an interannual average value of 58.85 µg·m^−3^) (Table 1), the increasing trends of which, meanwhile, were remarkably significant, and the growth rate of the former (with *β_slope_* = 0.367 and *Z_MK_* = 6.252) was greater than that of the latter (with *β_slope_* = 0.232 and *Z_MK_* = 3.767) (Table 2). In addition, there was a rapid increase in surface ozone concentration levels in the whole area of TJ from 2016 to 2017, which was especially more rapid over the non-built-up area, and then a slow increase in ozone concentration levels in Tianjin appeared, especially over the non-built-up area, which showed a slow fluctuating increase from 2017 to 2019, while over the built-up area, the slow increase slowed down even further from 2018 to 2019 (Table 1). Comparatively, surface ozone concentration in Tangshan (TS) was the lowest among the three regions (Beijing, Tianjin, and Tangshan) during the period from 2016 to 2019, and surface ozone concentration in its northern part was clearly higher than that of its southern part (see Figure 2). The surface ozone concentration over the built-up area of TS increased slowly with high significance (with *β_slope_* = 0.305 and *Z_MK_* = 5.702) over the four-year study period, and in the meantime, there was a slow fluctuating decrease in the ozone concentration from 2017 to 2019 (Table 1 and Table 2).

**Figure 3:**
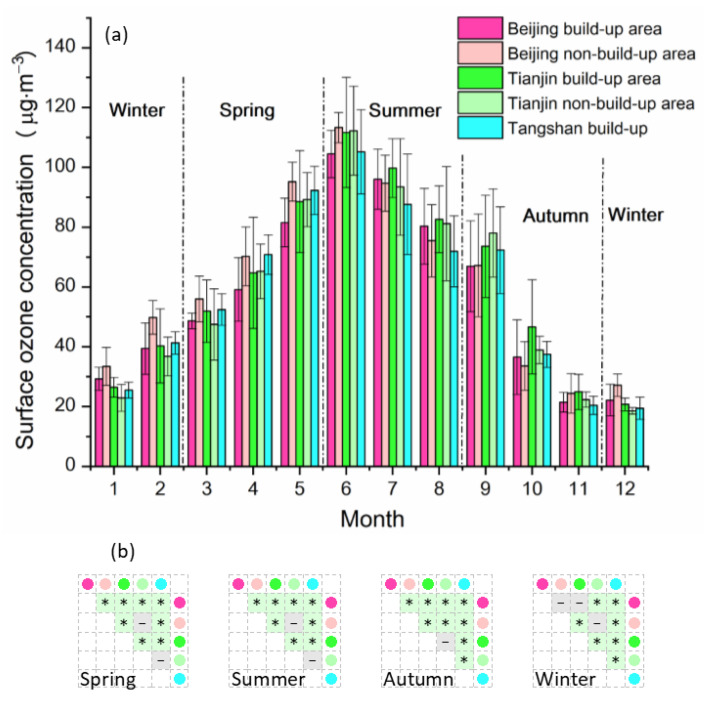
Monthly dynamics of maximum daily 8 h-averaged ozone concentrations (MDA8) in different seasons (**a**) and seasonal difference testing among subareas (**b**). * Significant difference (*p* < 0.01)—No significant difference (*p* > 0.1).

The multi-year seasonal and monthly variations in surface ozone concentration in the BJ-TJ-TS region from 2016 to 2019 exhibited a single-peak structure, as shown in Figure 3. It can be seen that the seasonal ozone concentration in this region displayed the relative order of summer > spring > autumn > winter. In spring, the ozone concentration in the non-built-up area of Beijing was the highest, while that in the built-up area of Beijing was the lowest. In summer, the maximum daily 8 h-averaged ozone concentration (MDA8) was generally high in all regions, reaching its annual maximum in June. July and August had the second- and fourth-highest ozone values of the year. Hence, high temperature and strong solar radiation in summer are conducive to the formation of ozone, and a low concentration of PM_2.5_ fine particulate matter can also play an important role in promoting ozone formation under certain meteorological conditions. Previous findings [29] indicated that there was a significant negative correlation between surface ozone concentration and PM_2.5_ concentration in Beijing and other related places (with PM_2.5_ concentration being comparatively lower in summer but relatively high in winter). In autumn, the MDA8 in the BJ-TJ-TS region rapidly decreased. In September, the MDA8 in the Beijing area decreased significantly, while those in Tianjin and the built-up area of Tangshan decreased slightly compared to their August values, which may be related to concentration changes in other preceding atmospheric pollutants, such as CO, SO_2_, and PM_2.5_ fine particulate pollutants [30]. From October to November and December, the MDA8 in the entire region decreased sharply. In autumn, the MDA8 in Tianjin was higher than that in Beijing and the built-up area of Tangshan. In October, the MDA8 values in the non-built-up areas of Beijing and Tianjin were higher than those in built-up areas. In winter, the MDA8 in all regions reached its lowest value in December, and the average MDA8 value in Beijing for the winter season was higher than that in Tianjin and the built-up area of Tangshan (see Figure 3b).

**Figure 4:**
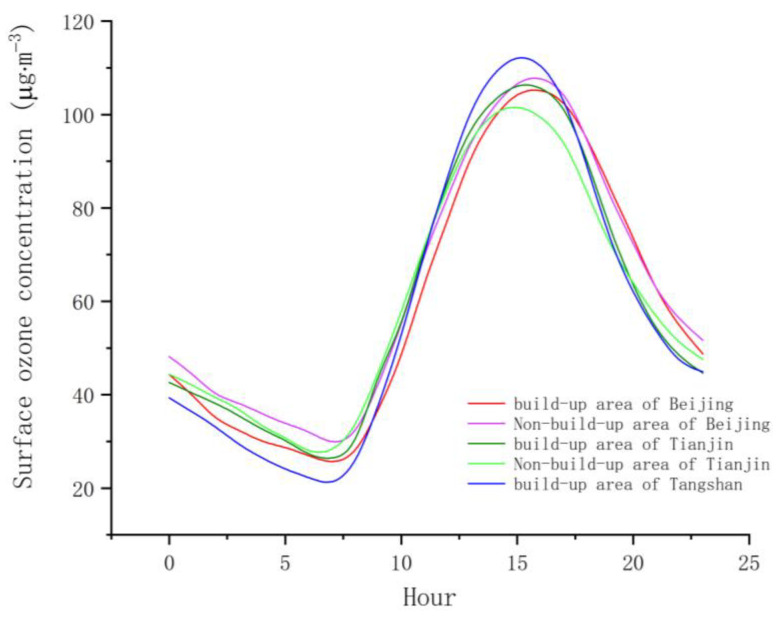
Diurnal variation curves of ozone concentration.

Figure 4 plots the diurnal variation in surface ozone concentration in the BJ-TJ-TS region from 2016 to 2019. The diurnal variation curves of surface ozone concentration are similar, although there are differences in terms of amplitude and slope, and maximum and minimum values occurred at slightly different times. The minimum ozone concentration occurred at about 7:00 in the morning, after which it steadily increased, reaching its peak between 15:00 and 16:00 and then decreasing. The minimum ozone concentration values of the five curves were very similar. The minimum in Tangshan was less than those in the other regions, while its maximum was the largest. Hence, the curve of Tangshan’s ozone concentration exhibited the greatest amplitude and change rate. The time of the maximum ozone concentration in the Beijing area was about one hour later than the peak times in Tianjin and Tangshan. In addition, the curves of Beijing’s and Tianjin’s built-up areas had greater amplitude than the curves of their corresponding non-built-up areas. This may be due to the stronger accumulation and consumption capacity of their built-up areas compared to their non-built-up areas [32].

### 3.2. Relationship between Ozone and Meteorological Factors

Meteorological factors play a very important role in the formation and transformation of ozone. For example, they influence changes in ozone concentration by influencing the diffusion of ozone precursors, atmospheric circulation, and the photochemical environment [33]. Variations in surface ozone concentration, especially involving the occurrence of high ozone concentration, the formation of its pollution process, the expansion of or reduction in its pollution scope, and the increase or decrease in its pollution degree, are all closely related to certain meteorological conditions due to the induction or control exerted by these conditions. The BJ-TJ-TS region, a densely populated area, is the very significant core urban agglomeration of China’s capital economic zone, i.e., the Beijing–Tianjin–Hebei (BJ-TJ-HB) zone [34], which is also known as one of the most important and developed integrated economic regions in China. Moreover, the livable, green, and sustainable development of the BJ-TJ-TS region plays a key and core role in leading the finely integrated regional development activities of the BJ-TJ-HB economy. Geographically, the BJ-TJ-TS region is dominated by plains and mountains, the latter of which are generally found in its north and west sections, with corresponding elevations of approximately 600–1500 m and 1200–2000 m. This region is located in the northeastern portion of the North China Plain and covers a relatively small area, the eastern boundary of which is along the coast of the Bohai Sea. It is part of the warm temperate semi-humid monsoon climate zone, with four distinct seasons. Interactions of its regional atmosphere with atmospheric circulations affected by large geographical features, such as the Tibetan Plateau, are very apparent, manifesting as meteorological “leeward slope” effects such as sinking air and lighter winds. In the context of the special location and underlying surface features of this region, since the formation of surface ozone and its pollution, the effects of its related accumulative increases or decreases, and the spatiotemporal characteristics of its changes are affected by causes such as the secondary synthetic transformation of atmospheric contaminants and regional transport of ozone, they are closely associated with the particular characteristics of certain meteorological factors (note that other factors, e.g., the emissions of industrial pollutants and automobile exhaust, are also linked to ozone) [13].

The association between the MDA8 values of surface ozone concentrations (which were derived from hourly ground monitoring observations) [35] and meteorological factors, including daily average air temperature, pressure, relative humidity, wind velocity, and sunshine duration (observed at the air quality observation and/or meteorological stations in the built-up and non-built areas of this study area on non-precipitation days from 2016 to 2019), is presented based on correlation coefficients in Table 3. Generally speaking, temperature exhibited the highest positive correlations with ozone concentration, with the second highest being negative correlations with pressure, and the third highest being for sunshine duration and wind velocity; very weak correlations (the lowest) were obtained for relative humidity. The correlation coefficients in terms of rank order were temperature > pressure > sunshine duration > wind velocity > relative humidity in the BJ-B, TJ-B, TJ-N, and TS areas and temperature > sunshine duration > pressure > relative humidity > wind velocity in the BJ-N area. Thus, as the temperature rises, along with enhancing evaporation from the surface (e.g., from soil and water) and vegetation evapotranspiration, in certain other meteorological conditions, the surface’s volatile emissions, such as from the soils and plants, including the key precursors of ozone, e.g., volatile organic compounds or VOCs such as isoprene (C5), monoterpenes (C10), and sesquiterpenes (C15), increase and further facilitate the formation of surface ozone [36,37]. The concentration of surface ozone is negatively correlated with pressure, which may be because, when a lower-pressure central area near the surface exists, the surface ozone over its surrounding high-pressure area in the atmospheric boundary layer is horizontally transported and accumulates in the lower-pressure central area so that its surface ozone concentration rises. On the contrary, if in a higher-pressure central area near the surface, its surface ozone diffuses to its surrounding low-pressure area so that its surface ozone concentration is reduced [38]. The process by which wind (wind velocity and direction) influences near-surface ozone is complex [39]. There is, in general, a weak correlation between surface ozone concentration and wind velocity, but under some conditions (such as a combination of high temperature, no rain, and moderately weak wind), the correlation between the two is strongly significant [40,41]. In addition, relative humidity in a range of 30–50% is favorable for the formation of surface ozone with high concentration, but too high or too low relative humidity is unfavorable [30,41].

**Figure 5:**
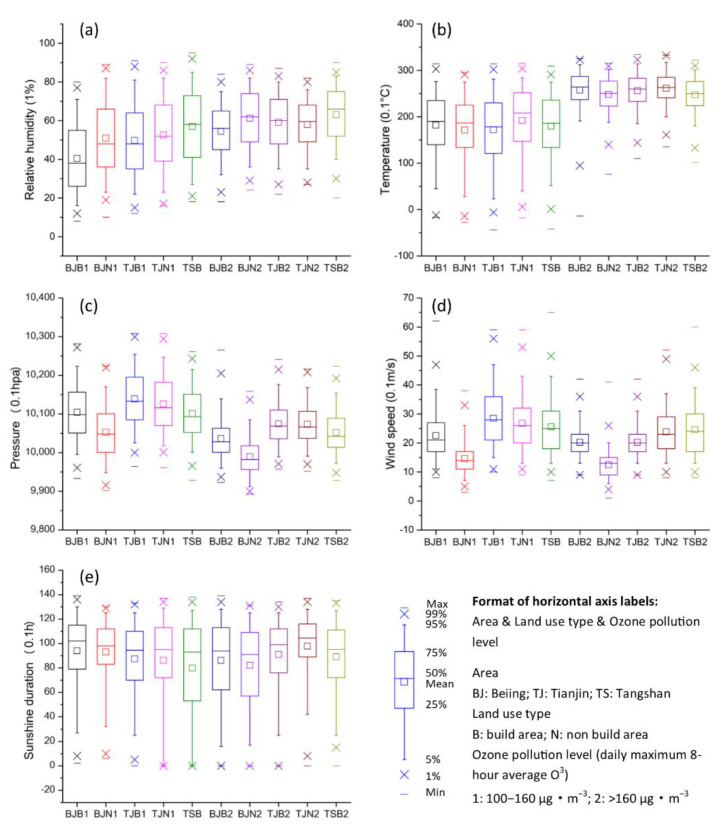
Meteorological factors associated with different MDA8 levels: (**a**) Relative humidity, (**b**) Temperature, (**c**) Pressure, (**d**) Wind speed, and (**e**) Sunshine duration. Note: The boxes encompass the 25th–75th percentiles and contain median lines.

Based on the classification grades of ozone pollution levels from China’s current air quality standard, which was established in 2012 (GB 3095-2012), we calculated the daily average air temperature, pressure, relative humidity, wind velocity, and sunshine duration for ozone pollution events occurring on days from 2016 to 2019, with the Grade I ozone pollution level defined as 100 μg·m^−3^ ≤ MDA8 < 160 μg·m^−3^ and Grade II defined as MDA8 ≥ 160 μg·m^−3^ (see Figure 5a–e). It was obvious that the meteorological conditions of high temperature, long sunshine duration, low atmospheric pressure, weak wind velocity, and certain relative humidity levels readily led to high-concentration ozone pollution. The daily average temperature on the days with Grade II ozone pollution was 7.4 °C higher than that on the days with Grade I ozone pollution, while the daily average relative humidity was 9% higher, the daily average pressure was 59 hPa lower, and the daily average wind velocity was 0.34 m·s^−1^ lower (as well as some high values of daily average sunshine duration with no apparent difference). A comparison of the geographical areas in which Grade I and Grade II ozone pollution events occurred during the 2016–2019 study period revealed that the daily average relative humidity levels and wind velocities in the BJ-B area were distinctly lower than those in the BJ-N area, whereas the daily average temperature and pressure values in the former were higher than those in the latter, and their daily average sunshine durations were not notably different. The relationships between the built-up and non-built-up areas of Tianjin (i.e., the TJ-B and TJ-N areas, respectively) were very similar to those of the Beijing area when Grade I and Grade II ozone pollution events occurred. In contrast, when Grade I and Grade II ozone pollution occurred in the Tianjin area, there was no significant difference in the daily average temperature, pressure, and relative humidity values between the TJ-B and TJ-N areas, while the daily average wind velocity in the TJ-B area was lower than that in the TJ-N area, and the sunshine duration in the built-up area was slightly lower than that in the non-built-up area. As for Grade I and Grade ozone pollution events occurring in the non-built-up area of Tangshan (TS) from 2016 to 2019, the daily average temperatures and relative humidity levels of the former were noticeably higher than those of the latter, the daily average pressure of the former was lower, and the daily sunshine durations were not significantly different.

### 3.3. Complexity and Uncertainty

Since the dynamics of surface ozone are mutually influenced via interference coupling with many factors, including the emissions of nitrogen oxides (NO_x_) and volatile organic compounds (VOCs) of ozone [28], the levels of fine particulate matter (e.g., PM_2.5_ and PM_10_) in the atmosphere [27], and meteorological conditions (such as atmospheric circulation patterns) [42,43], the relationships between surface ozone and its influencing factors are quite complex, making it very difficult to examine individual factors and independently analyze their effects on ozone. Therefore, correlation analyses between ozone and meteorological factors are also difficult, and there is unavoidable uncertainty in the analytical results [28]. For example, the correlation between ozone concentration and one meteorological parameter cannot be accurately presented (e.g., demonstrating a spurious-weak or strong correlation) [44] due to interactions with other meteorological parameters and/or non-meteorological parameters. This is acutely obvious, especially when coupled with special local climate and/or microclimate influences associated with extremely different geographical or underlying surface conditions such as urban areas and/or remote suburban areas [25], where there is directional transport (e.g., caused by the urban heat island effect) and/or exchanges (e.g., driven by urban and suburban wind eddies) of atmospheric pollutants between urban and surrounding areas, in which the relationships between ozone and various meteorological factors (such as temperature, precipitation, air pressure, wind velocity, and direction) become more complex and changeable [45,46].

Similarly, there are evident uncertain measures of surface ozone concentration in the built-up areas and non-built-up areas of this study region with values of ∆ = 21.561 µg·m^−3^ (within ∆*_β_* = 21.559 µg·m^−3^) and ∆ = 22.512 µg·m^−3^ (within ∆*_β_* = 22.507 µg·m^−3^), respectively, which shows that there were distinct differences in the distribution of surface ozone between the two classified areas from 2016 to 2019, and it may mean that the influences of the two types of underlying surfaces might be a very important cause of the regional differences in their surface ozone distribution (Table 4). In the meanwhile, there is also a similar situation in the Beijing and Tianjin areas, but the former (with ∆ = 20.351 µg·m^−3^ (∆*_β_* = 20.343 µg·m^−3^) of the built-up area, and ∆ = 22.215 µg·m^−3^ (∆*_β_* = 22.202 µg·m^−3^) of the non-built-up area) is located close to the interior area, whereas the latter (with ∆ = 23.014 µg·m^−3^ (∆*_β_* = 23.002 µg·m^−3^) of the built-up area, and ∆ = 23.32 µg·m^−3^ (∆*_β_* = 23.309 µg·m^−3^) of the non-built-up area) is near the Bohai Sea. Thus, their surface ozone distribution features have great regional differences, and there exists high consistency (of surface ozone distribution) between Tianjin’s and Tangshan’s built-up areas (because of the latter also being near the Bohai Sea) (Table 4).

Additionally, the networks of surface environment observation stations have their own deficiencies and are spatially sparse in the study area. Hence, the observation data volumes have, to some extent, small sample size limitations and representative problems in terms of geographical space [47]. These shortcomings could increase the difficulty of investigating problems, leading to certain, and even significant, impacts on the analysis or interpretation of such problems, i.e., increasing the uncertainties of research results [48,49]. As a result, it is necessary to fully understand the explicit or implicit actions of various possible elements in order to explore the spatiotemporal features of surface ozone, as well as the complicated and internal relationships of its interactions with associated factors, so as to provide further theoretical methods and support for basic information services for regional air pollution prevention and control.

**Table 4:** Uncertainty analysis for dynamic changes in surface ozone concentration in Beijing, Tianjin, and Tangshan from 2016 to 2019.

ID	Area		Average (µg·m^−3^)	Standard Deviation, *SD* (µg·m^−3^)	Uncertain Measure $\Delta =\sqrt{\Delta_{\alpha }^{2}+\Delta_{\beta }^{2}}$ (μg·m^−3^)	Component $\Delta_{\alpha }=\frac{SD}{\sqrt{n}}$ (μg·m^−3^)	Component $\Delta_{\beta \;}=\frac{SD}{\sqrt{3}}$ (μg·m^−3^)	Notes [50]
1	BJ	BJ-B	57.502	35.235	20.351	0.586	20.343	
2		BJ-N	63.888	38.455	22.215	0.754	22.202	
3	TJ	TJ-B	60.795	39.840	23.014	0.742	23.002	
4		TJ-N	59.161	40.373	23.320	0.710	23.309	
5	TS	TS-B	56.923	36.555	21.113	0.559	21.105	
6		TS-N	-	-	-	-	-	No data
7	BJ-TJ -TS	Built-up area	57.878	37.342	21.561	0.238	21.559	
8		Non-built-up area	60.928	38.984	22.512	0.467	22.507	

## 4. Conclusions

In this study, we discovered that the surface ozone concentration of the BJ-TJ-TS region from 2016 to 2019 exhibited an overall pattern of high values in the northwest and low values in the southeast, and there was an obvious difference between the built-up and non-built-up areas (especially in Beijing). The ozone levels noticeably increased in the BJ and TJ areas during the study period, while this upward trend was not evident in the TS area. The highest monthly average ozone values of all three areas occurred in the summer month of June, while the lowest monthly average levels occurred in the winter month of December. Their diurnal variation values reached a maximum value at approximately 3:00–4:00 p.m. and a minimum at approximately 7:00 a.m. It was clear that the meteorological conditions of high temperature, long sunshine duration, low pressure, weak wind velocity, and certain relative humidity levels could readily lead to high-concentration ozone pollution. Meanwhile, the daily average temperature on the days with Grade II ozone pollution was 7.4 °C higher than that on the days with Grade I ozone pollution, while the daily average relative humidity was 9% higher, the daily average pressure was 59 hPa lower, and the daily average wind velocity was 0.34 m·s^−1^ lower (as well as some high values of daily average sunshine duration with no apparent difference). At the same time, thanks to being mutually influenced by many related factors via interference coupling, the meteorological stress conditions, which were associated with spatiotemporal patterns and changes in surface ozone concentration and its pollution levels (including Grades I and II), resulted in certain regional differences, complexities, and relevant uncertainties during the 2016–2019 study period.

## Figures and Tables

**Table 1:** Dynamic changes in surface ozone average concentrations in study region from 2016 to 2019.

Year and Index	Built-up Area of Whole Region (µg·m^−3^)	Non-Built-up Area of Whole Region (µg·m^−3^)	Built-up Area of BJ (µg·m^−3^)	Non-Built-up Area of BJ (µg·m^−3^)	Built-up Area of TJ (µg·m^−3^)	Non-Built-up Area of TJ (µg·m^−3^)	Built-up Area of TS (µg·m^−3^)
2016	54.87	50.38	56.78	54.26	51.47	47.80	54.59
2017	57.37	63.06	55.29	64.81	61.26	61.90	57.55
2018	59.77	63.82	58.27	64.84	65.11	63.14	58.21
2019	59.40	62.50	58.37	62.43	65.72	62.54	56.55
Average value *	57.85	59.94	57.18	61.59	60.89	58.85	56.73
R^2^ *	0.841	0.342	0.474	0.400	0.835	0.632	0.286
Slope *	1.599	3.712	0.775	2.450	4.660	4.546	0.654
*p* *	0.083	-	-	-	0.086	-	-

Note: * Parameters of a model of linear regression (i.e., *Surface ozone concentration* = *Slope* × *Year* + *Constant*). Meanwhile, it must be noted that, although in the cases of the model, none passed tests of significance (*P*) level (comparatively with a level of less than 0.05, 0.01, or 0.001), except for two that passed with a *P* level less than 0.1 (i.e., with two levels of 0.083 and 0.086) in the whole study region and the built-up area of TJ, respectively, they would be mainly used to explain the variational tendencies of surface ozone concentration over the different subareas of Beijing (BJ), Tianjin (TJ), and Tangshan (TS).

**Table 2:** Trends of surface ozone concentration over Beijing, Tianjin, and Tangshan from 2016 to 2019 based on a 12-month moving-average time series.

ID and Testing	Period	Build-up Area of BJ (µg/m^−3^)	Non-Build-up Area of BJ (µg/m^−3^)	Build-up Area of TJ (µg/m^−3^)	Non-Build-up Area of TJ (µg/m^−3^)	Build-up Area of TS (µg/m^−3^)
1	January 2016 to December 2016	58.54	56.25	51.47	47.80	54.59
2	February 2016 to January 2017	58.99	56.61	51.89	48.44	54.34
3	March 2016 to February 2017	59.20	57.41	52.48	49.46	53.82
4	April 2016 to March 2017	59.17	58.42	53.76	51.26	53.89
5	May 2016 to April 2017	57.39	57.71	53.77	51.88	53.27
6	June 2016 to May 2017	56.89	58.97	54.40	53.25	52.18
7	July 2016 to June 2017	57.84	61.67	55.59	54.59	52.42
8	August 2016 to July 2017	56.94	62.68	57.24	56.28	53.67
9	September 2016 to August 2017	54.53	62.22	59.10	59.01	55.20
10	October 2016 to September 2017	54.44	63.19	60.41	61.34	56.17
11	November 2016 to October 2017	53.34	62.82	60.36	61.31	56.07
12	December 2016 to November 2017	54.38	64.10	60.81	61.68	56.74
13	January 2017 to December 2017	55.29	64.81	61.26	61.90	57.55
14	February 2017 to January 2018	55.97	65.74	61.50	62.10	58.03
15	March 2017 to February 2018	56.88	66.25	62.10	62.28	58.35
16	April 2017 to March 2018	56.73	65.85	62.16	62.56	58.75
17	May 2017 to April 2018	58.26	67.43	63.66	63.71	60.11
18	June 2017 to May 2018	57.29	66.38	63.37	63.54	60.74
19	July 2017 to June 2018	57.54	66.45	65.00	65.21	61.21
20	August 2017 to July 2018	56.67	64.70	65.09	64.85	59.68
21	September 2017 to August 2018	58.55	66.07	66.76	65.81	60.71
22	October 2017 to September 2018	57.87	65.04	64.83	63.10	58.65
23	November 2017 to October 2018	59.04	66.14	65.61	63.77	59.02
24	December 2017 to November 2018	58.45	65.06	65.32	63.27	58.48
25	January 2018 to December 2018	58.27	64.84	65.11	63.14	58.21
26	February 2018 to January 2019	57.59	63.93	64.70	62.57	57.88
27	March 2018 to February 2019	56.81	63.36	64.07	62.38	57.95
28	April 2018 to March 2019	57.32	63.41	64.56	62.33	58.53
29	May 2018 to April 2019	56.64	61.78	63.81	61.76	58.23
30	June 2018 to May 2019	56.96	61.71	64.82	62.30	59.44
31	July 2018 to June 2019	56.99	61.18	63.34	60.91	59.40
32	August 2018 to July 2019	58.04	62.21	65.03	62.86	62.95
33	September 2018 to August 2019	56.39	60.60	63.45	60.73	62.50
34	October 2018 to September 2019	59.27	63.53	65.90	62.36	65.30
35	November 2018 to October 2019	58.57	62.67	65.94	62.37	65.76
36	December 2018 to November 2019	58.60	63.00	65.89	62.51	65.90
37	January 2019 to December 2019	58.37	62.88	65.72	62.54	65.72
TMS estimate * MK testing *	*β_slope_*	0.026	0.082	0.367	0.232	0.305
	*Z_MK_*	0.942	1.386	6.252	3.767	5.702

Note: * In the Mann–Kendall (MK) trend test, the statistic *Z_MK_* was utilized to detect whether or not a trend exists in the time series of surface ozone concentration (these data were preprocessed by using a 12-month moving-average approach so as to remove seasonal effects) based on an assessment criterion, that is, on the basis of *α* (i.e., 10%, 5% or 1%) significance level; if the absolute value $\left|Z_{Mk}\right|>\left|Z_{\alpha /2}\right|$ with a threshold value of 1.65, 1.96, and 2.58, respectively, it indicates that the trend passes the significance test with a reliability confidence limit of 90%, 95%, and 99%, respectively. Meanwhile, the trend is upward (if *Z_MK_* > 0) or downward (if *Z_MK_* < 0), or there is no trend (*Z_MK_* = 0). Additionally, associated with the Theil–Sen median slope (TMS) estimator, a slope of $\beta_{slope}=median\left(\frac{X_{j}-X_{i}}{j-i}\right)\;\forall \;1\leq i<j\leq n$ (where *i* and *j* are natural numbers) [31] was calculated to present the quantitative change trends (namely, upward with *β_slope_* > 0, downward with *β_slope_* < 0, and no trend with *β_slope_* = 0) of surface ozone concentration time series.

**Table 3:** Correlations of surface ozone concentration with meteorological factors.

Meteorological Factor	BJ-B	BJ-N	TJ-B	TJ-N	TS
Average temperature (0.1 °C)	0.812 **	0.785 **	0.834 **	0.836 **	0.823 **
Average wind speed (0.1 m·s^−1^)	0.248 **	0.053	0.240 **	0.165 **	0.252 **
Average pressure (0.1 hPa)	−0.748 **	−0.361 **	−0.773 **	−0.769 **	−0.758 **
Average relative humidity (1%)	0.110 **	0.060 *	0.123 **	0.155 **	0.056 *
Sunshine duration (0.1 h)	0.385 **	0.408 **	0.496 **	0.500 **	0.408 **

** Correlation significant at the 0.01 level (double-tailed). * Correlation significant at the 0.05 level (double-tailed).
